# 2-(4-Fluoro­phen­yl)-1-phenyl-1*H*-imidazo[4,5-*f*][1,10]phenanthroline monohydrate

**DOI:** 10.1107/S1600536811026328

**Published:** 2011-07-09

**Authors:** S. Rosepriya, M. Venkatesh Perumal, A. Thiruvalluvar, J. Jayabharathi, R. J. Butcher, J. P. Jasinski, J. A. Golen

**Affiliations:** a PG Research Department of Physics, Rajah Serfoji Government College (Autonomous), Thanjavur 613 005, Tamilnadu, India; b Department of Chemistry, Annamalai University, Annamalai Nagar 608 002, Tamilnadu, India; c Department of Chemistry, Howard University, 525 College Street NW, Washington, DC 20059, USA; d Department of Chemistry, Keene State College, 229 Main Street, Keene, NH 03435-2001, USA

## Abstract

In the title compound, C_25_H_15_FN_4_·H_2_O, the fused ring system is essentially planar [maximum deviation of 0.0822 (14) Å]. The imidazole ring makes dihedral angles of 76.83 (7) and 32.22 (7)° with the phenyl group attached to nitro­gen and the fluoro­benzene group to carbon, respectively. The dihedral angle between the two phenyl rings is 72.13 (7)°. Inter­molecular O—H⋯N, O—H⋯F, C—H⋯F, C—H⋯O and C—H⋯N hydrogen bonds are found in the crystal structure.

## Related literature

For a related structure, see: Rosepriya *et al.* (2011 ▶). For technological and biological applications of related compounds, see: Liu *et al.* (2005 ▶); Bian *et al.* (2002 ▶).
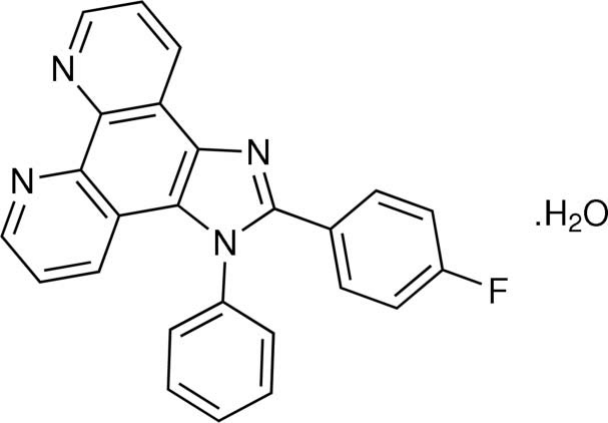


## Experimental

### 

#### Crystal data

C_25_H_15_FN_4_·H_2_O
*M* *_r_* = 408.43
Triclinic, 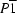

*a* = 9.0103 (10) Å
*b* = 10.0946 (9) Å
*c* = 11.3994 (9) Å
α = 86.987 (7)°
β = 75.950 (8)°
γ = 82.396 (8)°
*V* = 996.75 (17) Å^3^
*Z* = 2
Cu *K*α radiation
μ = 0.75 mm^−1^
*T* = 170 K
0.34 × 0.25 × 0.20 mm

#### Data collection

Oxford Diffraction Xcalibur Eos Gemini diffractometer
Absorption correction: multi-scan (*CrysAlis RED*; Oxford Diffraction, 2010 ▶) *T*_min_ = 0.784, *T*_max_ = 0.864
6315 measured reflections
3765 independent reflections
3382 reflections with *I* > 2σ(*I*)
*R*_int_ = 0.013

#### Refinement

*R*[*F* ^2^ > 2σ(*F* ^2^)] = 0.042
*wR*(*F* ^2^) = 0.122
*S* = 1.04
3765 reflections
288 parameters
H atoms treated by a mixture of independent and constrained refinement
Δρ_max_ = 0.20 e Å^−3^
Δρ_min_ = −0.20 e Å^−3^

### 

Data collection: *CrysAlis PRO* (Oxford Diffraction, 2010 ▶); cell refinement: *CrysAlis PRO*; data reduction: *CrysAlis RED* (Oxford Diffraction, 2010 ▶); program(s) used to solve structure: *SHELXS97* (Sheldrick, 2008 ▶); program(s) used to refine structure: *SHELXL97* (Sheldrick, 2008 ▶); molecular graphics: *ORTEP-3* (Farrugia, 1997 ▶) and *PLATON* (Spek, 2009 ▶); software used to prepare material for publication: *PLATON*.

## Supplementary Material

Crystal structure: contains datablock(s) global, I. DOI: 10.1107/S1600536811026328/hg5061sup1.cif

Structure factors: contains datablock(s) I. DOI: 10.1107/S1600536811026328/hg5061Isup2.hkl

Additional supplementary materials:  crystallographic information; 3D view; checkCIF report

## Figures and Tables

**Table 1:** Hydrogen-bond geometry (Å, °)

*D*—H⋯*A*	*D*—H	H⋯*A*	*D*⋯*A*	*D*—H⋯*A*
O1*W*—H1*W*⋯N10^i^	0.96 (3)	2.62 (3)	3.282 (2)	127 (2)
O1*W*—H1*W*⋯N13^i^	0.96 (3)	1.97 (3)	2.902 (2)	163 (3)
O1*W*—H2*W*⋯F4^ii^	0.92 (4)	2.41 (3)	3.159 (2)	139 (3)
C9—H9⋯F4^iii^	0.95	2.49	3.248 (2)	136
C14—H14⋯O1*W*^iv^	0.95	2.56	3.510 (2)	176
C19—H19⋯N3^v^	0.95	2.61	3.5209 (19)	162

Symmetry codes: (i) 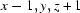; (ii) 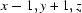; (iii) 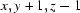; (iv) 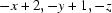; (v) 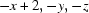.

**Fig. 1.**
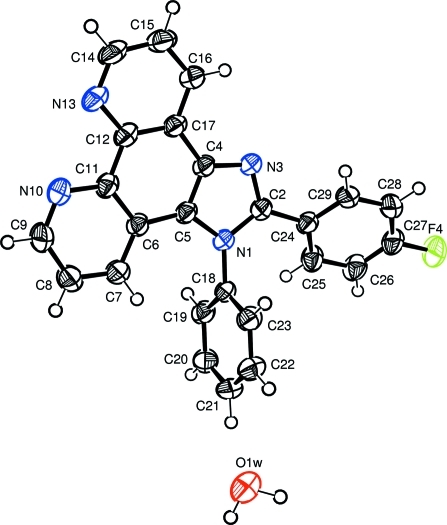
The molecular structure of the title compound, showing the atom-numbering scheme and displacement ellipsoids drawn at the 50% probability level. H atoms are shown as small spheres of arbitrary radius.

**Fig. 2.**
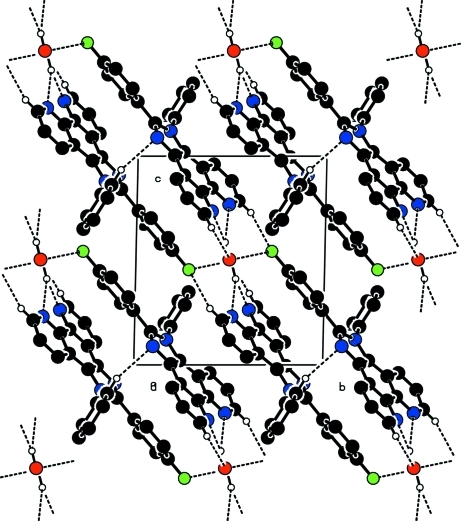
The packing of the title compound, viewed down the a axis. Dashed lines indicate hydrogen bonds. H atoms not involved in hydrogen bonding have been omitted.

## supplementary crystallographic information

### Comment

1,10-Phenanthroline has a rigid framework and possesses a superb ability to chelate many metal ions *via* two nitrogen donors, which show potential for technological applications, due to their high charge transfer mobility, bright light-emission and good electro- and photo-active properties (Liu *et al.*, (2005)). Phenanthroline ligands are particularly attractive species for developing new diagnostic and therapeutic agents that can recognize and cleave DNA. 1,10-Phenanthroline and its derivatives are commonly used as ligands in metal complexes (Bian *et al.*, (2002)). Rosepriya *et al.* (2011) have reported the crystal structure of 1,2-Diphenyl-1*H*-imidazo[4,5-*f*][1,10]phenanthroline. Since our group is doing research in organic light emitting devices (OLEDs), we are interested to use the title compound for synthesizing Ir(III) complexes and to study its photophysical properties.

The asymmetric unit of the title compound, C_25_H_15_FN_4_.H_2_O, contains a 2-(4-Fluorophenyl)-1-phenyl-1*H*-imidazo[4,5-*f*][1,10] phenanthroline molecule and a water solvent molecule. The fused ring system is essentially planar [maximum deviation of -0.0822 (14) Å for C15]. The imidazole ring makes dihedral angles of 76.83 (7) and 32.22 (7)° with the phenyl group attached to nitrogen and fluorobenzene group attached to carbon respectively. The dihedral angle between the phenyl ring and the benzene ring is 72.13 (7)°. Intermolecular O1W—H1W···N10, O1W—H1W···N13, O1W—H2W···F4, C9—H9···F4, C14—H14···O1W and C19—H19···N3 hydrogen bonds are found in the crystal structure (Table 1, Fig. 2).

### Experimental

To the pure 1,10-Phenanthroline-5,6-dione (2.10 g, 10 mmol) in ethanol (10 ml), aniline (0.91 g, 10 mmol), ammonium acetate (0.77 g, 10 mmol) and 4-fluorobenzaldehyde (1.1 g, 10 mmol) was over a period of about 1 h by maintaining the temperature at 333 K. The reaction mixture was refluxed for 7 days and then extracted with dichloromethane. The solid separated was purified by column chromatography using Benzene: Ethyl acetate as the eluent. Yield: 1.57 g (40%). Crystals suitable for X-ray diffraction studies were grown by slow solvent evaporation of a solution of the compound in dichloromethane.

### Refinement

H1W and H2W attached to O1W were located in a difference Fourier map and refined freely. Remaining H atoms were positioned geometrically and allowed to ride on their parent atoms, with C—H = 0.95 Å; *U*_iso_(H) = 1.2*U*_eq_(C).

### Crystal data

**Table:**

C_25_H_15_FN_4_·H_2_O	*Z* = 2
*M**_r_* = 408.43	*F*(000) = 424
Triclinic, *P*1	*D*_x_ = 1.361 Mg m^−^^3^
Hall symbol: -P 1	Melting point: 567 K
*a* = 9.0103 (10) Å	Cu *K*α radiation, λ = 1.54178 Å
*b* = 10.0946 (9) Å	Cell parameters from 4456 reflections
*c* = 11.3994 (9) Å	θ = 4.0–71.6°
α = 86.987 (7)°	µ = 0.75 mm^−^^1^
β = 75.950 (8)°	*T* = 170 K
γ = 82.396 (8)°	Block, pale-yellow
*V* = 996.75 (17) Å^3^	0.34 × 0.25 × 0.20 mm

### Data collection

**Table:**

Oxford Diffraction Xcalibur Eos Gemini diffractometer	3765 independent reflections
Radiation source: Enhance (Cu) X-ray Source	3382 reflections with *I* > 2σ(*I*)
graphite	*R*_int_ = 0.013
Detector resolution: 16.1500 pixels mm^-1^	θ_max_ = 71.7°, θ_min_ = 4.0°
ω scans	*h* = −10→10
Absorption correction: multi-scan (*CrysAlis RED*; Oxford Diffraction, 2010)	*k* = −12→11
*T*_min_ = 0.784, *T*_max_ = 0.864	*l* = −13→9
6315 measured reflections	

### Refinement

**Table:**

Refinement on *F*^2^	Primary atom site location: structure-invariant direct methods
Least-squares matrix: full	Secondary atom site location: difference Fourier map
*R*[*F*^2^ > 2σ(*F*^2^)] = 0.042	Hydrogen site location: inferred from neighbouring sites
*wR*(*F*^2^) = 0.122	H atoms treated by a mixture of independent and constrained refinement
*S* = 1.04	*w* = 1/[σ^2^(*F*_o_^2^) + (0.0685*P*)^2^ + 0.1908*P*] where *P* = (*F*_o_^2^ + 2*F*_c_^2^)/3
3765 reflections	(Δ/σ)_max_ = 0.001
288 parameters	Δρ_max_ = 0.20 e Å^−^^3^
0 restraints	Δρ_min_ = −0.20 e Å^−^^3^

### Special details

**Table:**

Geometry. Bond distances, angles *etc*. have been calculated using the rounded fractional coordinates. All su's are estimated from the variances of the (full) variance-covariance matrix. The cell e.s.d.'s are taken into account in the estimation of distances, angles and torsion angles
Refinement. Refinement of *F*^2^ against ALL reflections. The weighted *R*-factor *wR* and goodness of fit *S* are based on *F*^2^, conventional *R*-factors *R* are based on *F*, with *F* set to zero for negative *F*^2^. The threshold expression of *F*^2^ > 2σ(*F*^2^) is used only for calculating *R*-factors(gt) *etc*. and is not relevant to the choice of reflections for refinement. *R*-factors based on *F*^2^ are statistically about twice as large as those based on *F*, and *R*- factors based on ALL data will be even larger.

### Fractional atomic coordinates and isotropic or equivalent isotropic displacement parameters (Å^2^)

**Table:**

	*x*	*y*	*z*	*U*_iso_*/*U*_eq_	
F4	0.97668 (13)	−0.28116 (12)	0.54415 (10)	0.0786 (4)	
N1	0.92907 (12)	0.16988 (11)	0.12069 (9)	0.0359 (3)	
N3	1.18158 (12)	0.09894 (11)	0.08946 (10)	0.0391 (3)	
N10	1.01571 (15)	0.48776 (13)	−0.23133 (12)	0.0521 (4)	
N13	1.31915 (15)	0.39690 (13)	−0.26380 (11)	0.0501 (4)	
C2	1.04372 (15)	0.08612 (13)	0.15819 (11)	0.0369 (4)	
C4	1.15633 (15)	0.19490 (13)	0.00436 (11)	0.0368 (4)	
C5	1.00239 (15)	0.24120 (13)	0.02073 (11)	0.0358 (4)	
C6	0.94466 (15)	0.34434 (13)	−0.05427 (12)	0.0372 (4)	
C7	0.79054 (17)	0.40253 (15)	−0.03881 (14)	0.0457 (4)	
C8	0.75310 (18)	0.49897 (16)	−0.11884 (15)	0.0532 (5)	
C9	0.8689 (2)	0.53716 (17)	−0.21396 (16)	0.0584 (5)	
C11	1.05532 (16)	0.39312 (13)	−0.15231 (12)	0.0394 (4)	
C12	1.21950 (16)	0.34406 (14)	−0.17030 (12)	0.0400 (4)	
C14	1.46849 (18)	0.35781 (17)	−0.27695 (15)	0.0551 (5)	
C15	1.52874 (18)	0.26431 (17)	−0.20121 (15)	0.0529 (5)	
C16	1.42936 (16)	0.20624 (16)	−0.10893 (13)	0.0456 (4)	
C17	1.27071 (15)	0.24631 (14)	−0.09146 (12)	0.0380 (4)	
C18	0.76897 (14)	0.19088 (13)	0.18319 (11)	0.0353 (3)	
C19	0.66066 (16)	0.13798 (15)	0.13745 (13)	0.0433 (4)	
C20	0.50721 (17)	0.15814 (17)	0.19911 (14)	0.0515 (5)	
C21	0.46428 (17)	0.22966 (18)	0.30488 (15)	0.0545 (5)	
C22	0.57309 (18)	0.28226 (17)	0.34889 (14)	0.0540 (5)	
C23	0.72683 (17)	0.26361 (14)	0.28769 (13)	0.0441 (4)	
C24	1.01826 (15)	−0.00849 (13)	0.26178 (12)	0.0383 (4)	
C25	0.88803 (18)	−0.07485 (15)	0.29655 (13)	0.0458 (4)	
C26	0.87439 (19)	−0.16753 (16)	0.39112 (14)	0.0518 (5)	
C27	0.99130 (19)	−0.19174 (16)	0.45003 (14)	0.0525 (5)	
C28	1.12160 (18)	−0.12905 (18)	0.41907 (15)	0.0563 (5)	
C29	1.13416 (16)	−0.03709 (16)	0.32457 (14)	0.0483 (5)	
O1W	0.26297 (19)	0.49467 (15)	0.50499 (13)	0.0729 (5)	
H7	0.71305	0.37486	0.02691	0.0549*	
H8	0.64960	0.53925	−0.10958	0.0639*	
H9	0.84085	0.60287	−0.27007	0.0701*	
H14	1.53858	0.39588	−0.34194	0.0662*	
H15	1.63700	0.24125	−0.21342	0.0636*	
H16	1.46717	0.13990	−0.05757	0.0547*	
H19	0.69131	0.08871	0.06496	0.0519*	
H20	0.43144	0.12293	0.16887	0.0618*	
H21	0.35903	0.24259	0.34746	0.0654*	
H22	0.54251	0.33145	0.42142	0.0647*	
H23	0.80214	0.30036	0.31725	0.0529*	
H25	0.80786	−0.05634	0.25498	0.0549*	
H26	0.78593	−0.21339	0.41473	0.0621*	
H28	1.20088	−0.14836	0.46145	0.0675*	
H29	1.22349	0.00773	0.30171	0.0580*	
H1W	0.260 (3)	0.470 (3)	0.588 (3)	0.115 (9)*	
H2W	0.161 (4)	0.521 (3)	0.507 (3)	0.115 (10)*	

### Atomic displacement parameters (Å^2^)

**Table:**

	*U*^11^	*U*^22^	*U*^33^	*U*^12^	*U*^13^	*U*^23^
F4	0.0789 (7)	0.0849 (8)	0.0670 (7)	−0.0159 (6)	−0.0147 (5)	0.0469 (6)
N1	0.0323 (5)	0.0407 (6)	0.0329 (5)	−0.0076 (4)	−0.0042 (4)	0.0066 (4)
N3	0.0338 (5)	0.0466 (6)	0.0347 (6)	−0.0061 (5)	−0.0048 (4)	0.0066 (5)
N10	0.0500 (7)	0.0548 (7)	0.0476 (7)	−0.0085 (6)	−0.0071 (6)	0.0181 (6)
N13	0.0477 (7)	0.0549 (7)	0.0423 (7)	−0.0139 (6)	0.0011 (5)	0.0114 (5)
C2	0.0354 (7)	0.0406 (7)	0.0339 (6)	−0.0068 (5)	−0.0064 (5)	0.0039 (5)
C4	0.0339 (6)	0.0431 (7)	0.0327 (6)	−0.0092 (5)	−0.0049 (5)	0.0032 (5)
C5	0.0351 (7)	0.0405 (7)	0.0312 (6)	−0.0105 (5)	−0.0044 (5)	0.0036 (5)
C6	0.0367 (7)	0.0397 (7)	0.0351 (7)	−0.0090 (5)	−0.0071 (5)	0.0033 (5)
C7	0.0391 (7)	0.0484 (8)	0.0470 (8)	−0.0078 (6)	−0.0062 (6)	0.0098 (6)
C8	0.0433 (8)	0.0537 (9)	0.0601 (10)	−0.0023 (7)	−0.0123 (7)	0.0129 (7)
C9	0.0563 (9)	0.0576 (9)	0.0580 (10)	−0.0043 (7)	−0.0143 (7)	0.0240 (8)
C11	0.0428 (7)	0.0399 (7)	0.0351 (7)	−0.0103 (5)	−0.0071 (5)	0.0056 (5)
C12	0.0416 (7)	0.0430 (7)	0.0338 (6)	−0.0125 (6)	−0.0028 (5)	0.0029 (5)
C14	0.0451 (8)	0.0644 (10)	0.0483 (8)	−0.0177 (7)	0.0070 (7)	0.0100 (7)
C15	0.0373 (8)	0.0648 (10)	0.0515 (9)	−0.0111 (7)	0.0017 (6)	0.0001 (7)
C16	0.0380 (7)	0.0548 (8)	0.0419 (7)	−0.0089 (6)	−0.0046 (6)	0.0028 (6)
C17	0.0359 (7)	0.0444 (7)	0.0331 (6)	−0.0109 (5)	−0.0041 (5)	0.0004 (5)
C18	0.0319 (6)	0.0381 (6)	0.0333 (6)	−0.0071 (5)	−0.0036 (5)	0.0088 (5)
C19	0.0395 (7)	0.0519 (8)	0.0383 (7)	−0.0120 (6)	−0.0064 (5)	0.0036 (6)
C20	0.0367 (7)	0.0685 (10)	0.0508 (9)	−0.0165 (7)	−0.0106 (6)	0.0108 (7)
C21	0.0335 (7)	0.0713 (10)	0.0498 (9)	−0.0014 (7)	0.0024 (6)	0.0092 (7)
C22	0.0498 (9)	0.0608 (9)	0.0434 (8)	−0.0001 (7)	0.0012 (6)	−0.0046 (7)
C23	0.0432 (7)	0.0471 (7)	0.0413 (7)	−0.0075 (6)	−0.0076 (6)	−0.0007 (6)
C24	0.0384 (7)	0.0398 (7)	0.0328 (6)	−0.0035 (5)	−0.0029 (5)	0.0039 (5)
C25	0.0512 (8)	0.0472 (8)	0.0424 (7)	−0.0147 (6)	−0.0150 (6)	0.0086 (6)
C26	0.0563 (9)	0.0503 (8)	0.0488 (8)	−0.0194 (7)	−0.0087 (7)	0.0118 (7)
C27	0.0566 (9)	0.0513 (8)	0.0429 (8)	−0.0032 (7)	−0.0046 (7)	0.0181 (7)
C28	0.0437 (8)	0.0697 (10)	0.0507 (9)	−0.0003 (7)	−0.0105 (7)	0.0206 (8)
C29	0.0350 (7)	0.0579 (9)	0.0474 (8)	−0.0049 (6)	−0.0047 (6)	0.0143 (7)
O1W	0.0730 (9)	0.0857 (10)	0.0516 (8)	−0.0067 (7)	−0.0022 (6)	0.0075 (6)

### Geometric parameters (Å, °)

**Table:**

F4—C27	1.358 (2)	C18—C19	1.387 (2)
O1W—H1W	0.96 (3)	C19—C20	1.384 (2)
O1W—H2W	0.92 (4)	C20—C21	1.384 (2)
N1—C18	1.4386 (17)	C21—C22	1.378 (2)
N1—C2	1.3821 (17)	C22—C23	1.384 (2)
N1—C5	1.3883 (16)	C24—C29	1.396 (2)
N3—C4	1.3716 (17)	C24—C25	1.392 (2)
N3—C2	1.3164 (18)	C25—C26	1.383 (2)
N10—C11	1.3516 (19)	C26—C27	1.371 (2)
N10—C9	1.322 (2)	C27—C28	1.369 (2)
N13—C12	1.3524 (19)	C28—C29	1.378 (2)
N13—C14	1.325 (2)	C7—H7	0.9500
C2—C24	1.4721 (18)	C8—H8	0.9500
C4—C5	1.374 (2)	C9—H9	0.9500
C4—C17	1.4353 (19)	C14—H14	0.9500
C5—C6	1.4369 (19)	C15—H15	0.9500
C6—C11	1.4201 (19)	C16—H16	0.9500
C6—C7	1.407 (2)	C19—H19	0.9500
C7—C8	1.365 (2)	C20—H20	0.9500
C8—C9	1.388 (2)	C21—H21	0.9500
C11—C12	1.465 (2)	C22—H22	0.9500
C12—C17	1.408 (2)	C23—H23	0.9500
C14—C15	1.392 (2)	C25—H25	0.9500
C15—C16	1.367 (2)	C26—H26	0.9500
C16—C17	1.401 (2)	C28—H28	0.9500
C18—C23	1.3787 (19)	C29—H29	0.9500
			
H1W—O1W—H2W	102 (3)	C18—C23—C22	118.99 (14)
C2—N1—C18	126.14 (10)	C2—C24—C25	123.33 (13)
C5—N1—C18	127.06 (11)	C2—C24—C29	117.98 (13)
C2—N1—C5	106.32 (11)	C25—C24—C29	118.63 (13)
C2—N3—C4	104.84 (11)	C24—C25—C26	120.54 (15)
C9—N10—C11	117.97 (14)	C25—C26—C27	118.54 (16)
C12—N13—C14	118.04 (13)	F4—C27—C26	118.50 (15)
N1—C2—N3	112.21 (11)	C26—C27—C28	123.04 (15)
N1—C2—C24	125.07 (12)	F4—C27—C28	118.46 (15)
N3—C2—C24	122.72 (12)	C27—C28—C29	117.95 (15)
N3—C4—C5	111.71 (11)	C24—C29—C28	121.30 (14)
N3—C4—C17	126.82 (12)	C8—C7—H7	120.00
C5—C4—C17	121.46 (12)	C6—C7—H7	120.00
N1—C5—C6	131.96 (13)	C9—C8—H8	121.00
C4—C5—C6	123.11 (12)	C7—C8—H8	121.00
N1—C5—C4	104.91 (11)	N10—C9—H9	118.00
C5—C6—C11	116.29 (12)	C8—C9—H9	118.00
C7—C6—C11	117.27 (12)	N13—C14—H14	118.00
C5—C6—C7	126.43 (13)	C15—C14—H14	118.00
C6—C7—C8	119.59 (14)	C16—C15—H15	121.00
C7—C8—C9	118.83 (16)	C14—C15—H15	121.00
N10—C9—C8	124.07 (16)	C15—C16—H16	121.00
N10—C11—C12	116.99 (12)	C17—C16—H16	121.00
C6—C11—C12	120.77 (12)	C20—C19—H19	121.00
N10—C11—C6	122.22 (13)	C18—C19—H19	121.00
N13—C12—C11	117.77 (12)	C19—C20—H20	120.00
C11—C12—C17	120.63 (12)	C21—C20—H20	120.00
N13—C12—C17	121.60 (13)	C22—C21—H21	120.00
N13—C14—C15	123.90 (15)	C20—C21—H21	120.00
C14—C15—C16	118.82 (15)	C21—C22—H22	120.00
C15—C16—C17	118.81 (14)	C23—C22—H22	120.00
C4—C17—C12	117.64 (13)	C22—C23—H23	121.00
C4—C17—C16	123.59 (13)	C18—C23—H23	121.00
C12—C17—C16	118.76 (13)	C24—C25—H25	120.00
N1—C18—C19	119.61 (11)	C26—C25—H25	120.00
N1—C18—C23	118.92 (12)	C27—C26—H26	121.00
C19—C18—C23	121.47 (13)	C25—C26—H26	121.00
C18—C19—C20	118.93 (13)	C27—C28—H28	121.00
C19—C20—C21	119.92 (15)	C29—C28—H28	121.00
C20—C21—C22	120.50 (15)	C24—C29—H29	119.00
C21—C22—C23	120.18 (15)	C28—C29—H29	119.00
			
C5—N1—C2—N3	−0.42 (15)	C5—C6—C7—C8	−179.24 (14)
C5—N1—C2—C24	−179.84 (12)	C11—C6—C7—C8	1.8 (2)
C18—N1—C2—N3	−172.89 (12)	C5—C6—C11—N10	178.39 (13)
C18—N1—C2—C24	7.7 (2)	C5—C6—C11—C12	−2.95 (19)
C2—N1—C5—C4	0.35 (14)	C7—C6—C11—N10	−2.6 (2)
C2—N1—C5—C6	−178.18 (14)	C7—C6—C11—C12	176.11 (13)
C18—N1—C5—C4	172.74 (12)	C6—C7—C8—C9	0.0 (2)
C18—N1—C5—C6	−5.8 (2)	C7—C8—C9—N10	−1.4 (3)
C2—N1—C18—C19	−107.55 (15)	N10—C11—C12—N13	0.22 (19)
C2—N1—C18—C23	72.48 (17)	N10—C11—C12—C17	179.47 (13)
C5—N1—C18—C19	81.51 (17)	C6—C11—C12—N13	−178.51 (13)
C5—N1—C18—C23	−98.46 (16)	C6—C11—C12—C17	0.7 (2)
C4—N3—C2—N1	0.29 (15)	N13—C12—C17—C4	−179.27 (13)
C4—N3—C2—C24	179.73 (12)	N13—C12—C17—C16	1.9 (2)
C2—N3—C4—C5	−0.05 (14)	C11—C12—C17—C4	1.5 (2)
C2—N3—C4—C17	179.34 (13)	C11—C12—C17—C16	−177.36 (13)
C11—N10—C9—C8	0.8 (2)	N13—C14—C15—C16	1.5 (3)
C9—N10—C11—C6	1.3 (2)	C14—C15—C16—C17	−2.1 (2)
C9—N10—C11—C12	−177.42 (14)	C15—C16—C17—C4	−178.24 (14)
C14—N13—C12—C11	176.69 (13)	C15—C16—C17—C12	0.6 (2)
C14—N13—C12—C17	−2.6 (2)	N1—C18—C19—C20	179.50 (13)
C12—N13—C14—C15	0.9 (2)	C23—C18—C19—C20	−0.5 (2)
N1—C2—C24—C25	33.4 (2)	N1—C18—C23—C22	−179.07 (13)
N1—C2—C24—C29	−149.58 (14)	C19—C18—C23—C22	1.0 (2)
N3—C2—C24—C25	−146.01 (14)	C18—C19—C20—C21	−0.3 (2)
N3—C2—C24—C29	31.05 (19)	C19—C20—C21—C22	0.6 (3)
N3—C4—C5—N1	−0.20 (15)	C20—C21—C22—C23	−0.2 (3)
N3—C4—C5—C6	178.50 (12)	C21—C22—C23—C18	−0.6 (2)
C17—C4—C5—N1	−179.62 (12)	C2—C24—C25—C26	176.81 (13)
C17—C4—C5—C6	−0.9 (2)	C29—C24—C25—C26	−0.2 (2)
N3—C4—C17—C12	179.23 (13)	C2—C24—C29—C28	−177.09 (14)
N3—C4—C17—C16	−2.0 (2)	C25—C24—C29—C28	0.1 (2)
C5—C4—C17—C12	−1.4 (2)	C24—C25—C26—C27	0.4 (2)
C5—C4—C17—C16	177.37 (14)	C25—C26—C27—F4	178.94 (14)
N1—C5—C6—C7	2.4 (2)	C25—C26—C27—C28	−0.5 (2)
N1—C5—C6—C11	−178.60 (13)	F4—C27—C28—C29	−179.07 (15)
C4—C5—C6—C7	−175.86 (14)	C26—C27—C28—C29	0.3 (3)
C4—C5—C6—C11	3.10 (19)	C27—C28—C29—C24	−0.2 (2)

### Hydrogen-bond geometry (Å, °)

**Table:**

*D*—H···*A*	*D*—H	H···*A*	*D*···*A*	*D*—H···*A*
O1W—H1W···N10^i^	0.96 (3)	2.62 (3)	3.282 (2)	127 (2)
O1W—H1W···N13^i^	0.96 (3)	1.97 (3)	2.902 (2)	163 (3)
O1W—H2W···F4^ii^	0.92 (4)	2.41 (3)	3.159 (2)	139 (3)
C9—H9···F4^iii^	0.95	2.49	3.248 (2)	136
C14—H14···O1W^iv^	0.95	2.56	3.510 (2)	176
C19—H19···N3^v^	0.95	2.61	3.5209 (19)	162

Symmetry codes: (i) *x*−1, *y*, *z*+1; (ii) *x*−1, *y*+1, *z*; (iii) *x*, *y*+1, *z*−1; (iv) −*x*+2, −*y*+1, −*z*; (v) −*x*+2, −*y*, −*z*.
